# Arrangement of Azidomethyl Group in Lupinine Azide: Structural and Spectroscopic Properties

**DOI:** 10.3390/molecules30030582

**Published:** 2025-01-27

**Authors:** Kymbat Kopbalina, Dmitrii Pankin, Mikhail Smirnov, Niyazbek Ibrayev, Dastan Turdybekov

**Affiliations:** 1 Department of Physics and Nanotechnology, Buketov Karaganda University, Universitetskaya 28, Karaganda 100024, Kazakhstan; 2 Center for Optical and Laser Materials Research, St. Petersburg State University, Ulianovskaya 5, 198504 St. Petersburg, Russia; dmitrii.pankin@spbu.ru; 3 Faculty of Physics, St. Petersburg State University, Universitetskaya Nab. 7/9, 199034 St. Petersburg, Russia; m.smirnov@spbu.ru; 4 Institute of Molecular Nanophotonics, Buketov Karaganda University, Universitetskaya 28, Karaganda 100024, Kazakhstan; niazibrayev@mail.ru; 5 Department of Physics, Abylkas Saginov Karaganda Technical University, Ave. Nazarbayev 56, Karaganda 100027, Kazakhstan; turdas@mail.ru

**Keywords:** lupinine, azides, IR absorbance, NMR, spectroscopy, DFT

## Abstract

Quinolizidine derivatives are an important class of substances that are used in the pharmaceutical industry. In previous studies, the synthesis of these substances is carried out using lupinine azide (IUPAC: 1-(azidomethyl)octahydro-2H-quinolizine), which is often used to obtain new biologically active compounds. In this regard, its structural characterization is critically important. In this work, the conformational diversity of the molecular structure of this compound has been studied. It is shown that the structure with the axial position of the methyl azide group contains a number of low-energy conformer states with energies higher than the ground state by 0.15–0.60 kcal/mol. Such structural ambiguity should manifest itself in the chemical reactions and biological activity of lupinine azide. The spectroscopic properties of the conformers were studied by modeling chemical shifts for carbon and hydrogen atoms, as well as by simulating IR absorption spectra. An analysis of the most specific spectroscopic features of all of the conformers was carried out. Based on the correlation of the theoretical results and the experimental spectroscopic data, a conclusion was made for the first time regarding the most probable conformational states in the solution.

## 1. Introduction

The quinolizidine derivatives are an important class of substances used in the pharmaceutical industry [1]. Currently, they are considered promising substances for the treatment of diseases such as Alzheimer’s disease [1] as well as an agent for the treatment of arrhythmia [2,3]. However, their applicability is limited by their possible negative effects [2]. To understand the possible biological effect, a key issue is to establish a structure-versus-biological-activity correlation. Establishing such correlations requires experimental studies, including the use of model systems as well as theoretical modeling of the interaction of a molecule with a biological environment—molecular docking. The latter approach is especially promising, since it allows for avoiding labor-intensive and complex experiments and clearly shows the interaction of a biomolecule with a protein environment, accelerating the processes of drug discovery [4] and testing while simultaneously reducing the cost of the future drug and making it more accessible.

In this regard, it is critically important to study the structure of the biomolecule and develop methods for its characterization. The advanced method of theoretical study of biomolecules involves quantum-chemical calculations within the framework of the density functional theory, which allows for determining the stable and transition states of the molecule with high accuracy and reliability. Spectroscopic methods act as characterization methods that are very sensitive to the structure of organic substances. Among them, a special place is occupied by vibrational spectroscopy methods—IR absorption and Raman spectroscopy as well as nuclear magnetic resonance (NMR) spectroscopy. The advantages of optical vibrational spectroscopy methods are their sensitivity to the structure of the substance, their non-destructive effects and their relative ease of implementation as well as the possibility of continuous monitoring and the use of portable devices [5]. The advantages of the NMR spectroscopy method include the control of the structure at the atomic level and the high specificity with respect to the structure of the hydrogen arrangement [6]. In this regard, these methods are often used to characterize various synthesized structures. A comparison of the obtained experimental data with the results of the theoretical calculations allows us to obtain a complete interpretation of the experiment and shed light on the structure–property correlations.

At the moment, the structure of the closely related compounds lupinine and epilupinine is relatively well studied. Theoretical studies of the structures of lupinine and epilupinine, as well as their conformational isomers, can be found in [7,8,9]. Among the experimental methods aimed at characterizing the structure, one can highlight IR absorption spectroscopy [10], NMR spectroscopy on 1H and 13C nuclei [11,12] and X-ray diffraction [13,14].

At the same time, the work [15] notes that lupinine is a precursor for a possible two-stage synthesis of azidomethyl quinolizidine derivative (Lupinine azide. IUPAC: 1-(azidomethyl)octahydro-2H-quinolizine [15]), which opens up opportunities for the synthesis of biologically active quinolizine triazoles, which are important for the pharmacological industry [1].

To date, the study of azidomethyl quinolizidine derivatives has been carried out only experimentally using IR absorption, NMR spectroscopy and mass spectrometry; see, for example [15]. There are no references in the literature to studies devoted to the structure and ordering of the azidomethyl part, which is very flexibly connected to the bicycle. From a fundamental point of view, the study of the mutual ordering of the bicycle and the azidomethyl part in the ground and high-energy stable states and their population upon heating can be useful for controlling the biological activity of the compound or the derivatives based on it.

In addition, the identification of the arrangement of the azidomethyl moiety plays an important role in understanding how such molecular crystals are organized. This effect is closely related to the vibrational and NMR properties [16]. The importance of studying the arrangement of the azide moiety in azide derivative molecules for refining the crystal structure or cluster structure has been demonstrated in a number of theoretical (see, e.g., [17,18,19]) and experimental studies (see, e.g., [20,21]).

In this regard, an important task is the theoretical study of the structure of stable conformers and the spectroscopic characteristics of azidomethyl quinolizidine derivatives. In the future, this will contribute to understanding the structure of these compounds and finding their spectroscopic characteristics, general and specific, for various isomeric states.

In this work, the problem of the theoretical study of the structure of lupinine azide and its stable states, as well as the determination of the corresponding structure–spectrum correlations, is solved. Particular attention is paid to the study of the ordering of the azidomethyl part in the structure of stable states. Taking into account the practical and experimental possibility of using vibrational and NMR spectra to control the structure of substances, both in the in-line control of pharmaceutical production and during the synthesis of promising compounds, in this work they are studied theoretically and compared with experimental data obtained in other studies. 

## 2. Results and Discussion

### 2.1. Structural Peculiarities

**Figure 1:**
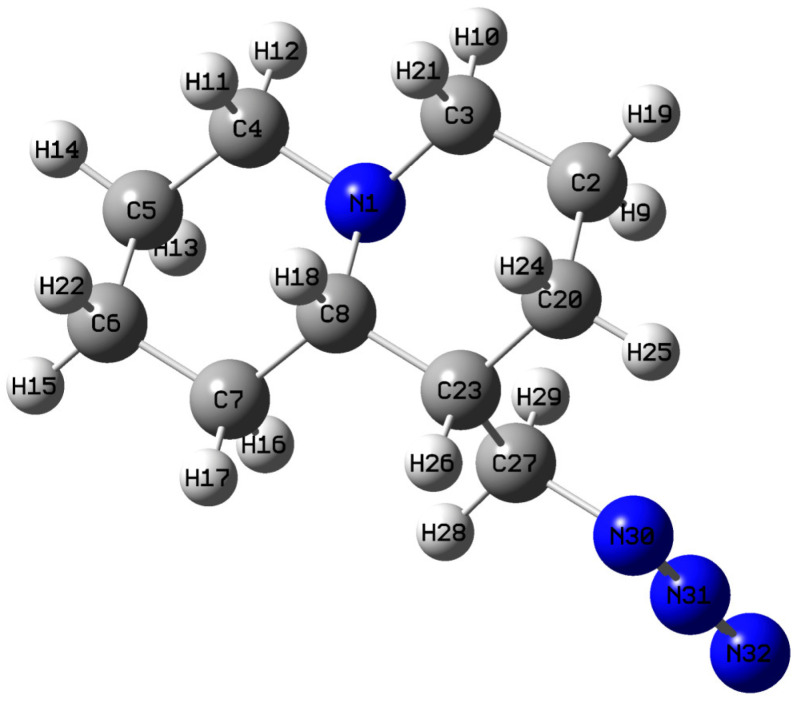
The optimized, most stable structure of lupinine azide (QAGAP1). Here, and subsequently for the other conformers, the light grey, the dark grey and the dark blue colors are used for the hydrogen, carbon and nitrogen atoms, respectively. The atomic labels shown here will be used later for other conformers.

In terms of composition, the structural difference between lupinine from the methyl azide quinolizidine derivative (MAQD) considered in this work (see Figure 1) is the presence of an OH group instead of an azide (NNN) group. The small size of the OH group results in a relatively weak change in energy during its rotation. The MeOH group can be in both the axial (lupinine) and equatorial (epilupinine) positions according to X-ray structural analysis data [13,14].

It should be noted that the spatial extent of the azide group, as well as the significant charge on the nitrogen atoms, determine the correlated ordering of the bicycle and the azide group in the chloroform (Chl) solvent.

In this regard, the experimental data from [13] for the lupinine molecule, in which the OH group was replaced by an azide group, was taken as the initial geometry for the theoretical study of the structure. This choice is associated with the previously conducted syntheses involving lupinine azide [15]. The result of the optimization of such a structure is shown in Figure 1. As can be seen, the quinolizidine has the azide group in axial positions. Further, for this structure, the notation QAGAP (derived from quinolizidine with the azide group in the axial position) will be used. Accordingly, we will designate the most stable state (the ground state) as QAGAP1. Such a molecule has the conformational flexibility associated with a change in the dihedral angles of C_B_C_B_C_Me_N_A_ and C_B_C_Me_N_A_N_A_, where the subindices B, Me and A signify belonging to a bicycle, methyl group and azide group, respectively.

Table 1 presents the values of the bond lengths and angles for QAGAP1. Due to the lack of experimental data for the methyl azide derivative and methyl azide, they are compared with experimental data for the lupinine molecule [13,19]. Nevertheless, it can be seen that for part of the bicycle, the calculation predicts values quite close to those observed in practice. In general, the ratios between different types of bonds are preserved. Thus, for the nitrogen-carbon bicyclic skeleton, the C-N bond lengths are in the range of 1.468–1.476 Å, whereas at the boundary of the methylene and azide groups they are 1.489 Å, which correlates with the lower Mulliken charge on the nitrogen atom of the azide group and greater charge heterogeneity. The longest bonds are C-C single bonds, the length of which varies in the range of 1.523–1.549 Å. The smallest values are predicted for the C2-C3 (1.523 Å) and C4-C5 (1.524 Å) bonds, and the longest for the C8-C23 (1.549 Å), C23-C20 (1.536 Å) and C7-C8 (1.535 Å) bonds. Thus, the presence of a methyl azide group leads to an elongation of the C-C bonds around the atom to which the addition occurs (see Table 1). The shortest non-hydrogen bonds are present in the azide group; these are the N30=N31 (1.225 Å) and N31≡N32 (1.133 Å) bonds. The obtained bond length values are in the ranges characteristic of double and triple bonds, respectively. These values are comparable with those obtained earlier in the calculation for methyl azide in [19,22]. The largest linear angles in the bicycle are associated with nitrogen atoms. The azide group differs little from the linear one, which is manifested in linear and dihedral angles close to 180°.

#### 2.1.1. Rotational Conformers

##### External Rotation of Methyl Azide Group

**Figure 2:**
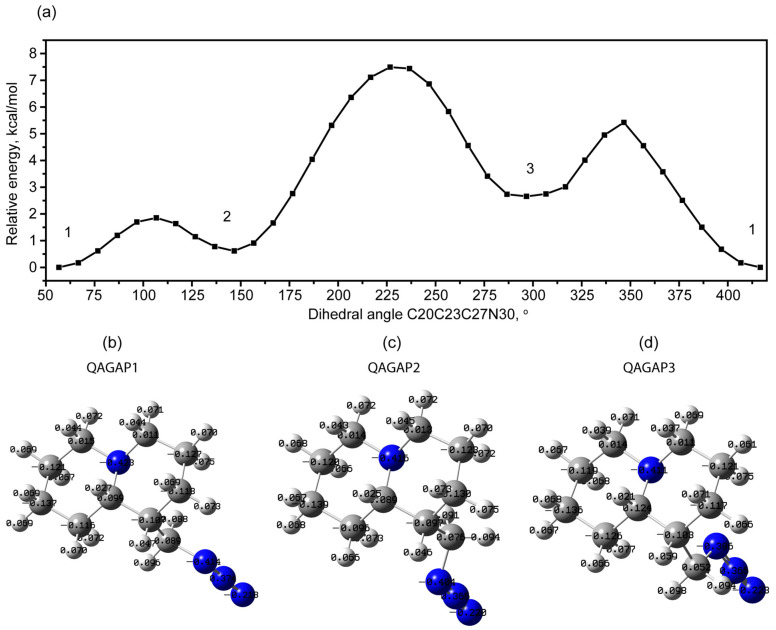
Potential energy scan with changing dihedral angle C20C23C27N30 (**a**). Optimized QAGAP1 (**b**), QAGAP2 (**c**) and QAGAP3 (**d**) structures with Mulliken charges on atoms.

When studying the conformational states of the molecule, low-energy conformational isomers associated with external and internal rotation in the methyl azide radical were found. Figure 2a shows a scan of the dependence of the potential energy on the change in the dihedral angle C20C23C27N30. The change in energy is shown relative to the energy of the most stable state, shown in Figure 1 and Figure 2b. This is the external rotation of the radical. In this scan, in addition to the ground state, two more stable states with higher energy can be distinguished: 2 (+0.62 kcal/mol) and 3 (+2.66 kcal/mol). Hereinafter in the text, these states will be designated QAGAP2 (Figure 2c) and QAGAP3 (Figure 2d), respectively. The estimated values of the potential barriers for the transition from QAGAP1 to QAGAP2 and from QAGAP1 to QAGAP3 in a chloroform solution are 1.85 and 5.42 kcal/mol, respectively.

**Figure 3:**
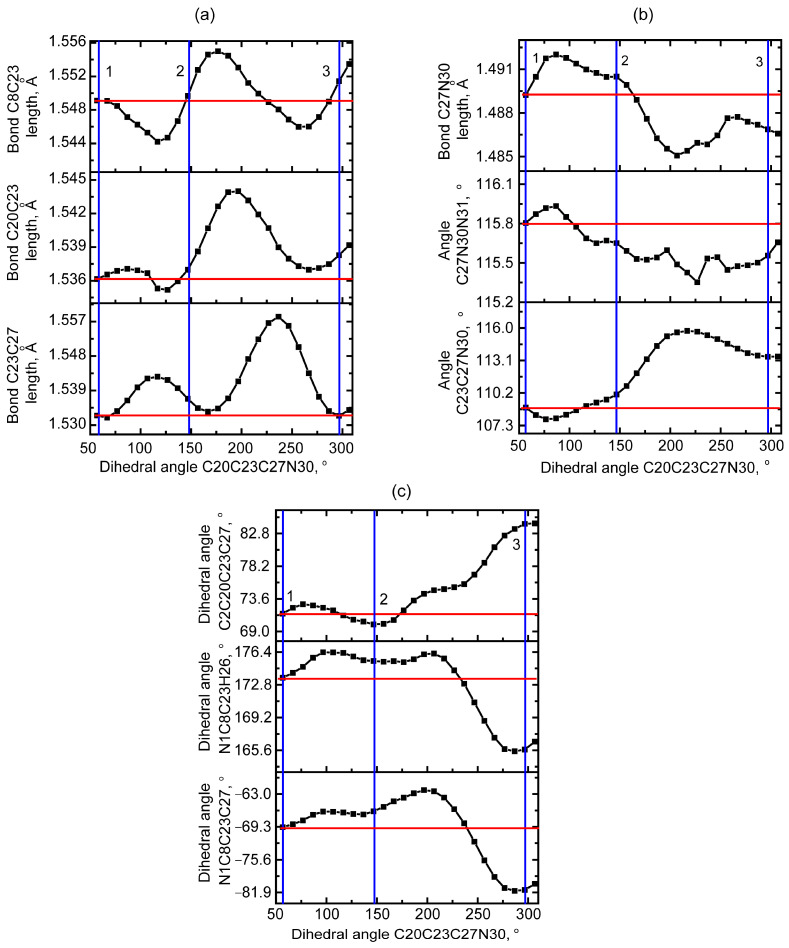
Dependencies of selected bond lengths (**a**,**b**), plane angles (**b**) and dihedral angles (**c**) on dihedral angle C20C23C27N30 during PES. The vertical blue lines corresponds to the optimized values of dihedral angle C20C23C27N30 of QAGAP1 (**1**), QAGAP2 (**2**) and QAGAP3 (**3**) states. The horizontal red lines corresponds to the optimized geometrical parameter value of QAGAP1.

When considering the structural features of these higher-energy states, a number of changes associated with the bicycle ring to which the methyl azide group is attached can be noted (Figure 3). For the optimized QAGAP2 and QAGAP3 structures, the values of the dihedral angles C20C23C27N30 are 145.6° and 296.7°. For optimized QAGAP2 and QAGAP3, the values of the dihedral C20C23C27N30 angles are 145.6 and 296.7°, respectively. For both states, a longer bond length in the bicycle for the C23 tertiary carbon atom can be noted. In the case of QAGAP2, the C23-C27 bond is also lengthened. At the same time, the C27-N30 bond between the methyl and azide groups is elongated in QAGAP2 and compressed in the QAGAP3 structure. A feature of the ordering of the methyl azide group in these states is a greater stretching of the methyl azide group with a distance from the conditional plane of the bicycle, which is manifested in a simultaneous decrease in the angle C27N30N31 and an increase in C23C27N30. Much more significant changes in the bicycle ring, to which the methyl azide group is attached, occur in the dihedral angles and amount to values from 8 to 12° in modulus in the QAGAP3 structure and from 1.4 to 3.3° in the QAGAP2 structure. This correlates with the relative energy values of the structures.

##### Internal Rotation of Methyl Azide Group

**Figure 4:**
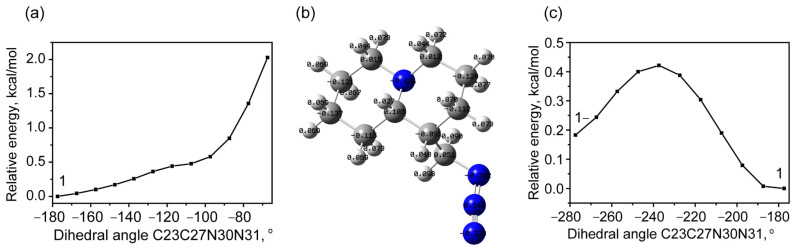
The potential energy as a function of the dihedral angle C23C27N30N31 changes counterclockwise (**a**), and the found higher-energy stable state QAGAP1- (**b**) during the clockwise dihedral angle C23C27N30N31 change in PES (**c**). For brevity, the states of QAGAP1 and QAGAP1- are denoted as 1 and 1- in (**a**,**c**). The QAGAP1- structure with corresponding Mulliken charges is shown in (**b**).

Internal rotation in the methyl azide group is associated with a change in the dihedral angle C23C27N30N31. No stable state was found during the counterclockwise rotation (Figure 4a), which can be associated with increasing repulsion as the azide tail and bicycle approach each other. At the same time, during the opposite motion (Figure 4b,c), associated with the distancing of these groups, a low-energy stable state appears, the energy of which is only 0.15 kcal/mol higher than that of QAGAP1 (Figure 4c). Let us designate this structure as QAGAP1-. The corresponding value of the dihedral angle C23C27N30N31 is 78.1°. The proximity of energies and a low potential barrier (approximately 0.42 kcal/mol) determine approximately equal thermodynamic probabilities of the QAGAP1 and QAGAP1- states.

**Figure 5:**
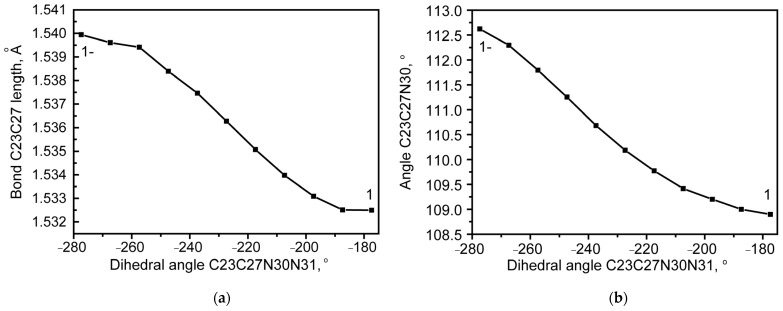
The changes in the length of the C23-C27 bond (**a**) and the planar C23C27N30 angle (**b**) when scanning with respect to the dihedral angle C23C27N30N31.

In the structure of QAGAP1-, as in the structure of QAGAP2, the deviations of the dihedral angles from their values in the ground state structure are very small. With an accuracy of 0.1° for the dihedral angles C2C20C23C27, N1C8C23H26 and N1C8C23C27, these deviations are equal to 0, 0.3 and 0.1°. At the same time, a significant increase in the length of the C23-C27 bond can be noted (Figure 5a), along with which a small increase in the planar C23C27N30 angle occurs.

**Figure 6:**
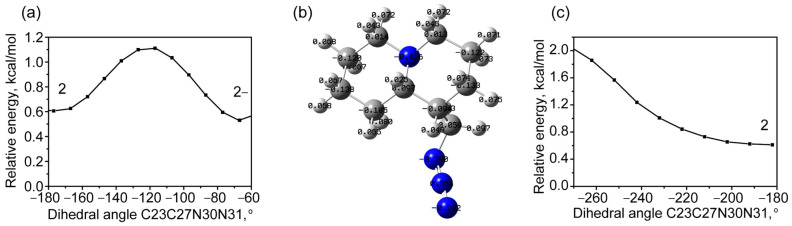
Potential energy scan with clockwise change of C23C27N30N31 dihedral angle (**a**); structure of QAGAP2- with corresponding Mulliken charges (**b**); potential energy scan with counterclockwise change of C23C27N30N31 dihedral angle (**c**). For brevity, states of QAGAP2 and QAGAP2- are denoted as 2 and 2-.

A qualitatively similar situation is observed for scanning with respect to the variation of the dihedral angle C23C27N30N31 when starting from the QAGAP2 structure. When rotating clockwise to a value of −180°, the presence of a minimum is noted, which is approximately 0.60 kcal/mol higher than the ground state (Figure 6a). It should be noted that the transition to this state is accompanied by a rotation of the dihedral angle C20C23C27N30 to 152.2°. Taking into account the direction of the rotation, this state is called QAGAP2- (Figure 6b).

When rotating counterclockwise (see Figure 6c), i.e., when the methyl azide group approaches the bicycle, a gradual increase in energy occurs; a stable state is absent when turning by 90°. As 90° is exceeded, a change in the dihedral angle begins with a gradual transition to QAGAP2-.

**Figure 7:**
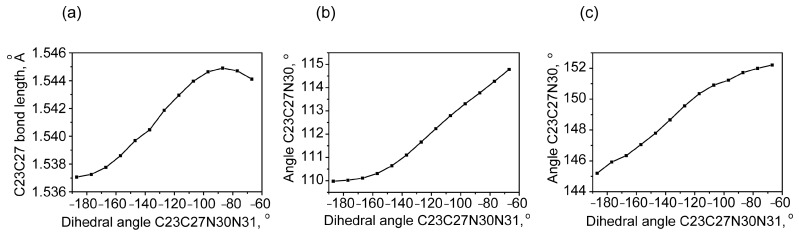
The change in the length of the C23-C27 bonds (**a**), the planar angle C23C27N30 (**b**) and the dihedral angle when scanning, with a change in the dihedral angle C23C27N30N31 (**c**).

During the transition from the QAGAP2 to the QAGAP2- state, a relatively small change in the dihedral angles C2C20C23C27, N1C8C23H26, N1C8C23C27 occurs within 0.3, 0.8 and 0.1° in absolute value. The most significant changes occur in the vicinity of the C23-C27 bond. They are associated with the shortening of the C23-C27 bond, a decrease in the planar angle C23C27N30 and the dihedral angle C20C23C27N30; see Figure 7.

**Figure 8:**
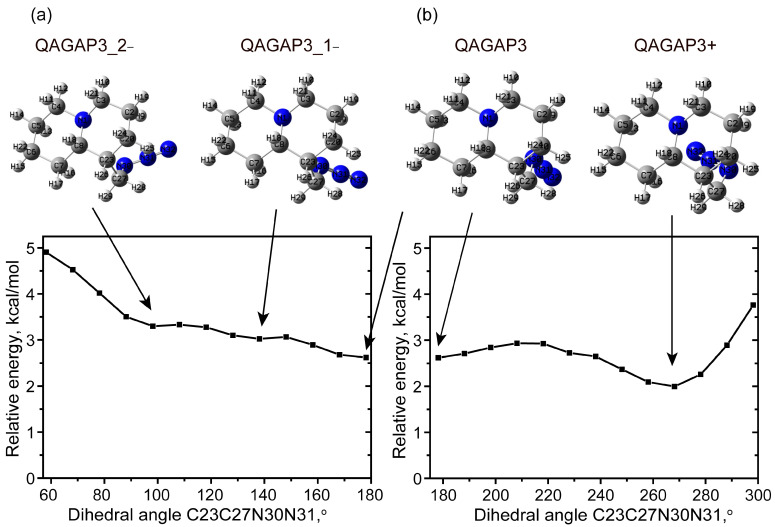
Potential energy scan with respect to clockwise (**a**) and counterclockwise (**b**) changes in dihedral angle C23C27N30N31.

It should be noted that the presence of the higher-energy rotational conformers, revealed by the scan of the dihedral angle C23C27N30N31 when starting from the QAGAP3 state, is also predicted. The dihedral angles C23C27N30N31 for QAGAP_3_1-, QAGAP3_2- and QAGAP3+ are approximately 138, 98 and 268 (−92)°, respectively. The potential energy scans are shown in Figure 8. The estimate of the relative population of the different rotamers, obtained from Boltzmann statistics, gives a total value of approximately 1% for the stable states, as shown in Figure 8.

Thus, the main contribution to the formation of bands in the vibrational spectra will be made by the QAGAP1, QAGAP1-, QAGAP2 and QAGAP2- rotamers. Their vibrational and NMR spectra will be discussed further.

### 2.2. NMR Properties

In the calculated and experimental 13C spectrum, higher chemical shifts have C8, C3 and C4 carbon atoms surrounding the electronegative N atom in the bicycle and C27 atom, through which the bicycle binds to the azide group; see Table 2. This correlates with the calculated Mulliken charges shown in Figure 2b. A somewhat lower chemical shift value is noted for the C23 tertiary carbon atom. The smallest chemical shift value is predicted for the C2 atom. For the C7, C5, C20 and C6 atoms, an intermediate chemical shift value is predicted in the range between that for the C2 and C23 atoms. Table 2 also shows the chemical shift values for the Boltzmann-weighted ensembles of the two (QAGAP1, QAGAP1-) and four (QAGAP1, QAGAP1-, QAGAP2, QAGAP2-) lowest-energy states. These were within a few percent of one another.

**Figure 9:**
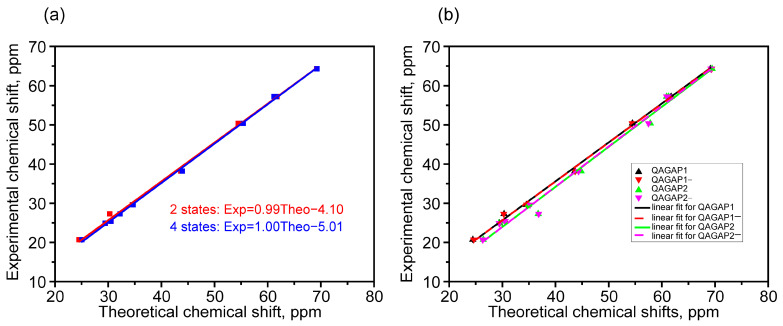
Linear fit in case of 2 (red) and 4 (blue) states taken into account (**a**), linear fit of individual states QAGAP1 (black), QAGAP1- (red), QAGAP2 (green) and QAGAP2- (purple) (**b**).

In general, when compared with the experiment, the calculation overestimates the chemical shift values on carbon nuclei by approximately constant +4.1 and +5.04 ppm (see Figure 9a). After applying the scaling procedure, the discrepancy in the absolute value decreased to 1.35 ppm. It can also be noted that there is a linear dependence between the calculation and the experiment results. For the most part, it is associated with the contributions of the most stable states QAGAP1 and QAGAP1-, the total share of which is estimated at approximately 70% within the Boltzmann statistics.

The relatively large contribution of these states was indirectly reflected in the Pearson’s R coefficient values for the linear approximation of unscaled chemical shifts for individual states (see Figure 9b). For the QAGAP1, QAGAP1-, QAGAP2 and QAGAP2- states, these coefficients were 0.99905, 0.99899, 0.99455 and 0.99493, respectively.

The most significant differences between the predicted chemical shifts in the QAGAP1, QAGAP1-, QAGAP2 and QAGAP2- structures were observed for the C27 and C20 atoms. In the case of the C27 atom, this can presumably be explained by the mutual arrangement of N30 atom interacting with H25 atom in QAGAP1 and QAGAP1- structures. The distance between H25 and N30 is 2.573 Å, which indicates the formation of a hydrogen bond. When passing to QAGAP2 and QAGAP2-, this bond disappears, which is consistent with the lower chemical shift of the H25 atom. At the same time, another hydrogen bond is formed between the H26 and N30 atoms, and simultaneously the chemical shift for the C27 atom increases. For the C20 atom, the calculation predicts an increase in the chemical shift when passing from QAGAP1, QAGAP1- to QAGAP2, QAGAP2-, which correlates with a larger negative Mulliken charge for the C20 atom in QAGAP2 and QAGAP2-.

It should be noted that when moving from QAGAP1, QAGAP1- to QAGAP2, QAGAP2-, significant changes also occur in the proton spectrum, namely, there is an increase in the chemical shifts for the H16 and H24 atoms, as well as a simultaneous decrease for the H9 and H25 atoms. The chemical shift for the H26 atom changes more complexly, which may be due to the influence of hydrogen atoms in the H28 and H29 positions, as well as the positively charged N31 atom.

Thus, the signals from the C20 and C27 nuclei can be used in practice to differentiate the two groups. The first consist of the structures QAGAP1 and QAGAP1-, and the second consist of the structures QAGAP2 and QAGAP2-. Further differentiation within each group can be carried out based on the signals from the protons in the methyl group at the boundary of the bicycle and the azide tail (H28 and H29). Their chemical shifts are much larger due to the immediate proximity of the electronegative N30 compared to the chemical shifts of the protons in the bicycle. The signal from the H26 proton, which is very specific for the states under consideration (see Table 1), can also be used for differentiation purposes.

### 2.3. IR Absorption Spectra Modelling

**Figure 10:**
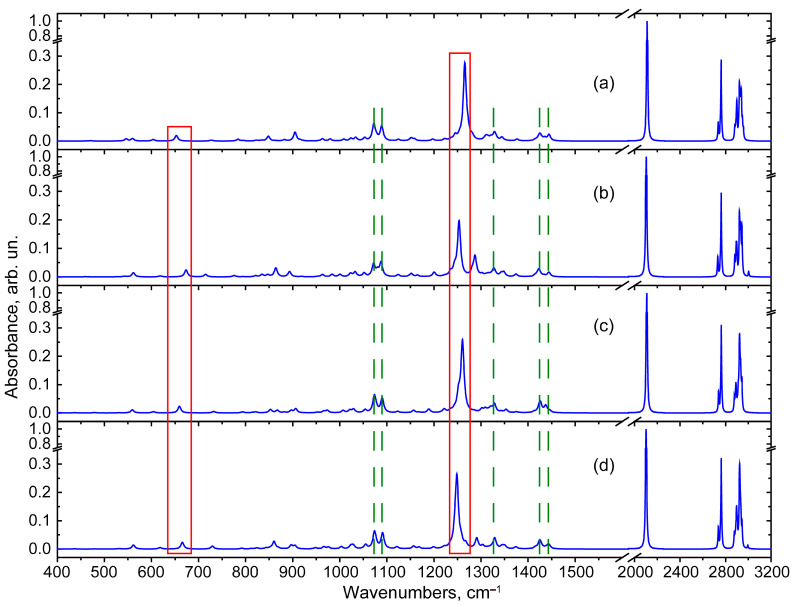
The calculated IR absorbance spectra for the different states: QAGAP1 (**a**), QAGAP1- (**b**), QAGAP2 (**c**), QAGAP2- (**d**). The vertical green dotted lines and red rectangles correspond to the regions of the less and more structure-sensitive vibrational modes.

In order to reveal the features sensitive to the conformational state of the molecule in the standard FTIR absorption spectrum, computer simulations of the vibrational IR-active modes were performed. Due to practical reasons and the spectroscopic limitations of commonly used FTIR spectrometers, the spectra in the region of 400–4000 cm^−1^ were discussed. In this section, our goal was to identify the high- and medium-active IR vibrational modes that are common to all states of lupinine azide, as well as the modes specific to each of the individual states: QAGAP1, QAGAP1-, QAGAP2 and QAGAP2-. The calculated IR absorption spectrum in the region of 400–3200 cm^−1^ is shown in Figure 10. The spectral region was reduced due to the absence of vibrational modes in the region above 3200 cm^−1^.

**Figure 11:**
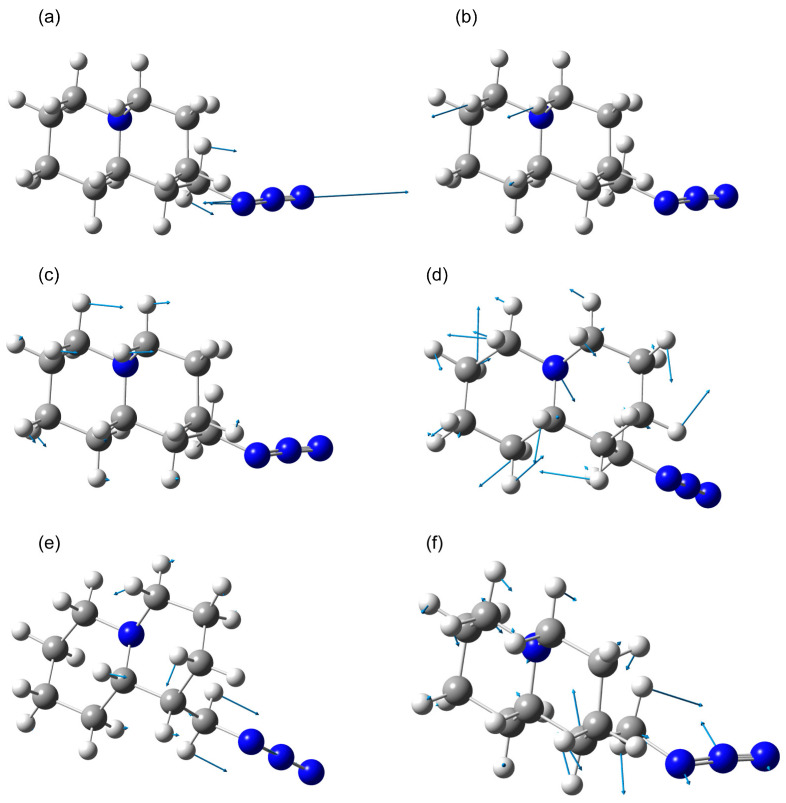
Examples of atomic displacements in vibrational modes of QAGAP1 with frequencies of 2110 (**a**), 2762 (**b**), 1328 (**c**), 1073 (**d**), 1266 (**e**) and 653 (**f**) cm^−1^.

In the calculated scaled IR absorption spectrum, the peak near 2100 cm^−1^ exhibits the highest activity. This is close to the experimentally observed peak in the IR absorption spectrum at 2096 cm^−1^ for lupinine azide in [15]. The calculated atomic displacements in this vibrational mode are localized in the azide part and have an antisymmetric character (see Figure 11a). A similar type of atomic displacement was noted for a similar mode in the calculations of methyl azide and formyl azide compounds in [19]. It should be noted that this mode is relatively weakly sensitive to the structural type. Thus, for QAGAP1, QAGAP1-, QAGAP2 and QAGAP2-, the calculation predicts frequencies of 2110, 2103, 2108 and 2101 cm^−1^, respectively. Moreover, for cases in which the azide group is directed perpendicular to the conventional plane of the bicycle (states QAGAP1 and QAGAP2), the calculation predicts higher values of the frequency of this vibrational mode (2110 and 2108 cm^−1^), which indicates a weaker interaction of the azide group and the bicycle. On the other hand, when the azide group is approximately parallel to the conventional plane of the bicycle (states QAGAP1- and QAGAP2-), the frequency values are lower (2103 and 2101 cm^−1^, respectively).

In the region of large wavenumbers (the range of 2725–2975 cm^−1^), the peaks with stretching vibrations involving hydrogen atoms are noted. At the same time, in the range of 2725–2775 cm^−1^, the vibrational modes of C-H bonds appear, in which carbon atoms surround the N1 atom in the bicycle. Thus, in the intense mode of 2761 cm^−1^, the predominant contributions are given by the in-phase vibrations v(C4-H11) and v(C3-H21), and in the less active mode of ~2735–2738 cm^−1^, the main contribution is given by the vibration v(C8-H18). In the experimental spectrum [15], these modes correspond to bands with maxima at 2744 and 2762 cm^−1^. Since the bonds in which these vibrations are localized are isolated from the methyl azide group, the corresponding spectral peaks are practically insensitive to the position of this group relative to the bicycle, which is manifested in close frequency values for different QAGAP states, as well as in a qualitatively similar result in the calculation for lupinin [8]. These bands, if sufficiently active in the spectrum, can be used as characteristic bands of the bicycle.

The spectral range of 2875–2975 cm^−1^ can be divided into two parts. The first part, 2875–2925 cm^−1^, includes modes with a mixed character of atomic displacements of the types v(C-H) and v_sym_(CH_2_). In the region around 2925 cm^−1^ in the calculated spectrum, a contribution of modes with an antisymmetric character of atomic displacements in the methylene groups (v_asym_(CH_2_)) with different phase relationships is noted. In the calculated IR absorbance spectrum with 4 cm^−1^ HWHM broadening, there are main bands with maxima at 2891–2898 and 2923 cm^−1^. However, in the experiment [15], only one peak is noted around 2804 cm^−1^. The reason for the possible discrepancies may be related to the strong anharmonicity of hydrogen vibrations [23] as well as the presence of hydrogen bonds not taken into account in the calculation. The latter can significantly change the contour of the bands of stretching vibrations v(C-H).

In the region of smaller wavenumbers compared to the peak at 2100 cm^−1^, all of the peaks have frequencies below 1500 cm^−1^, which is typical for saturated hydrocarbons. The highest-frequency peaks among them are those of deformation hydrogen vibrations, which appear in the calculated spectra as a doublet with maxima at 1425–1448 cm^−1^. Despite its relatively low activity, this doublet is located quite locally, which allows it to be considered separately from the rest of the spectrum. Its spectral position is less affected by a possible calculation error. The atomic displacements for the low-frequency component of the doublet are delocalized over the entire bicycle, which leads to a spread of frequencies of 1423–1425 cm^−1^ for different states. At the same time, the high-frequency component of the doublet contains a contribution from vibrations of the CH_2_ group in the methyl azide part. This leads to some variability of the maximum at 1437–1445 cm^−1^. Another fairly stable peak, distinguishable in the calculated IR absorption spectra for all four states, is the peak at about 1328 cm^−1^, which is associated with wagging vibrations in the methylene groups of the bicycle. The largest atomic shifts in this vibrational mode are localized in the w(H11C4H12) and w(H21C3H10) groups; see Figure 11c. Another characteristic spectral feature of the bicycle is the doublet, with frequencies of 1073 and 1089 cm^−1^, in which, along with the twisting modes localized in the methylene groups, the C-N bonds stretching localized in the bicycle also participate. An example of atomic shifts in the 1073 cm^−1^ mode is shown in Figure 10d. The localization of these modes in the bicycle and their high polarity, due to the contributions of v(C-N), lead to their relatively high IR activity and frequency stability.

In the conformers under consideration, the dependence of the spectrum on the structure, associated with the ordering of the methyl azide part, is manifested in a fairly intense band of ~1248–1270 cm^−1^. Its characteristic feature is associated with large displacements of the methyl azide part atoms. They include stretching vibrations of the N30=N31 bond, mixed with wagging vibrations w(H29C27H28), as well as with weaker hydrogen vibrations, delocalized over the bicycle (see Figure 11d). In the course of this study, it was found that the frequency of this mode is 1266, 1254, 1261 and 1249 cm^−1^ for QAGAP1, QAGAP1-, QAGAP2 and QAGAP2-, respectively. For this mode, one can note the frequency shift patterns similar to those established for the peak around 2100 cm^−1^: the frequencies of QAGAP1 and QAGAP1- are higher than the frequencies of QAGAP2 and QAGAP2-, and the frequencies of QAGAP1 and QAGAP2 are higher than the corresponding frequencies of QAGAP1- and QAGAP2-. In an experimental study of lupinine azide, this mode corresponded to a peak at 1269 cm^−1^ [15]. A similar peak was noted in the spectra of compounds with an azide group [18,20].

When considering the vibrational modes localized in the azide group, the presence of a mode with a deformation character of atomic displacements localized in the azide group, δ(NNN), was noted. Its frequency was 653, 673, 659 and 665 cm^−1^ for the structures QAGAP1, QAGAP1-, QAGAP2 and QAGAP2-, respectively. The nature of the dependence of the frequency of this deformation mode on the structure differs from the behavior of the stretching modes: higher frequency values are characteristic of the structures QAGAP1- and QAGAP2-, and lower ones are characteristic of QAGAP1 and QAGAP2. The presence of a similar mode has been reported in earlier studies of the vibrational properties of organic compounds with azide groups—both experimental (see, for example, [20,24]) and theoretical (see, for example, [18,20,25]) studies.

Thus, when considering the vibrational properties of individual states, it was proposed to identify the presence of certain conformers based on modes with atomic displacements that are localized in the methyl azide group. While vibrational modes with atomic displacements delocalized over the bicycle can be considered as characteristic bicycle modes, they, as a rule, weakly depend on the orientation of methyl azide.

## 3. Methodological Part

The quantum-chemical calculations within the DFT framework were performed using the Gaussian G09W Rev. D.01 software (Gaussian Inc., Wallingford, CT, USA) [26]. The B3LYP [27,28] was chosen as the exchange-correlation functional. Pople’s split-valence triple-zeta basis set, supplemented with polarization functions of the form 6-311G(2d,p), was used [29]. This approach allows for the accurate prediction of the structural, electronic and vibrational properties of various molecules, which was noted in previous studies [23,30,31,32]. In view of the importance of lupinine azide for pharmacology, it was necessary to take into account the interactions of the molecule with the environment (accounting for the influence of the solvent). This approach significantly brings the theoretical model closer to the situation in practice and in an actual experiment. In this work, the Chl was considered as the solvent. Chl was chosen due to its use in studying the structure of a substance through NMR spectroscopy and the choice of a solvent in the synthesis of bioactive molecules. To be able to compare the influence of different environments, they were taken into account implicitly using the polarizable continuum model (PCM) [33,34,35].

The molecular structure was optimized until the convergence criteria for the maximum and root-mean-square values of the atomic displacements and forces were met. The conformations corresponding to the higher-energy minima of the potential surface were identified using a relaxed scan with a 15° change in the dihedral angle. The stability of the minima found was checked for the absence of imaginary vibrational frequencies. The transition states were identified as saddle points of the potential surface with one imaginary frequency. The vibrational frequencies, IR absorption and Raman scattering spectra were calculated at the next stage. A scaling factor of 0.958 was used for a more accurate correlation between the calculated frequencies and the experimentally observed peaks. It was determined based on the fitted linear relationship between the theoretically calculated peaks in this work and the corresponding experimentally observed peaks (1269, 2096, 2744 and 2762 cm^−1^) in [15]. The correspondence between the experimental and theoretical peaks, as well as the assignments, were discussed further in Section 2.3. For practical purposes, unless otherwise stated, the calculated spectra presented in this work are given in scaled frequency values. In the calculated spectra, the half-width at half-maximum (HWHM) was assumed to be 4 cm^−1^. The calculated structural and vibrational properties for important stable states are demonstrated in the Supporting Information. The visualization of the optimized structures, as well as the vectors of atomic displacements in the vibrational modes, was performed using the GaussView 6.0.16 program (Gaussian Inc., Wallingford, CT, USA).

In addition, for practical purposes of characterizing the structure of the substance, the NMR properties were calculated using the Gauge-Independent Atomic Orbital (GIAO) approach [36,37,38]. The chemical shifts were calculated according to (1) [6]:

Ch. Shift = I_ref_ − I_sample_, (1)

where I_ref_ = $\frac{\sum_{i=1}^{3}I_{\mathrm{ii},\mathrm{ref}}}{3}$ isotropic value of magnetic shielding tensor with I_ij,ref_ (where *i*, *j* = 1…3) components for reference and I_sample_ = $\;\frac{\sum_{i=1}^{3}I_{\mathrm{ii},\mathrm{sample}}}{3}$ isotropic value of magnetic shielding tensor with I_ij,sample_ (where *i*, *j* = 1…3) components for sample. In this work, tetramethylsilane (TMS) was used as a reference sample. Its NMR properties were calculated in the same way as those of the substance being studied. For TMS, the calculated proton and carbon isotropic values of the magnetic shielding tensor were 31.963 and 184.144 ppm. For the practical purposes of identifying different states, theoretical chemical shifts were scaled. The scaling procedure is discussed in the text.

To estimate the probability of the state (p_i_), the Boltzmann distribution was used. According to this, the $p_{i}=\frac{e^{-\frac{E_{i}}{\mathrm{kT}}}}{Z}$, where Z = $\overset{M}{\underset{j=1}{\sum }}e^{-\frac{E_{j}}{\mathrm{kT}}}$ is the canonical partition function (normalization denominator), k—Boltzmann constant, T = 298.15K, and E_i_ and E_j_—energy of ith and jth state.

## 4. Conclusions

The structural features of the lupinine azide molecule (1-(azidomethyl)octahydro-2H-quinolizine)), which is an important precursor for the further synthesis of biologically active quinolizidine derivatives, are studied in this work. The case of the axial addition of the methyl azide group (the so-called QAGAP structure) is considered. The study of conformers obtained by external and internal rotation of the methyl azide radical led to the discovery of several conformers with energies higher than the ground state energy by 0.15–0.6 kcal/mol. Changing the orientation of the methyl azide group, such as by heating, can change the population ratio of such states. This may lead to changes in the chemical reactivity and biological activity.

In this study, the spectroscopic characteristics of conformers were also studied. This information will help to detect these states by NMR spectroscopy and IR absorption spectroscopy. Carbon atoms with the NMR signals that are the most sensitive to structural changes have been identified. The proton-related NMR spectrum of lupinine azide was interpreted for the first time on the basis of theoretical calculations and compared with the available experimental data. It is shown that the hydrogen atoms of the methyl group in the methyl azide part are especially promising for the structure characterization. An assessment of their relative contributions to the spectrum of different conformers was made, and the states giving the maximum contributions were identified. The frequencies of the vibrational modes and the IR absorption spectral curves for individual conformers were also studied. It has been shown that the modes in which the largest atomic amplitudes are localized in the methyl azide group are the most useful for identifying the location of the methyl azide radical relative to the bicycle. According to our calculations, the frequencies of such modes lie in the approximate ranges of 2100, 1250 and 650 cm^−1^. A set of spectral peaks characteristic of the bicyclic skeleton was also identified.

The obtained theoretical estimates of the probabilities of detection of the various conformers of the lupinine azide molecule based on their characteristic spectral lines can facilitate the experimental identification of these conformers.

## Figures and Tables

**Table 1:** The selected parameters of *QAGAP1* for the most stable found geometry (ground state). The bond length values are rounded to the 3rd decimal place. The plane and dihedral angles are rounded to the 1st decimal place. The experimental data were taken from [13,19].

Bonds	Calc. Bond Length (Å) in QAGAP1	Exp. Bond Length (Å) in Lupinine	Angles	Calc. Angles (°) in QAGAP1	Exp. Angles (°) in Lupinine
N1C3	1.468	1.472	N1C3C2	112.8	112.7
C3C2	1.523	1.502	C3C2C20	110.4	109.9
C2C20	1.529	1.513	C2C20C23	111.0	110.7
C20C23	1.536	1.533	C20C23C8	109.9	110.5
C23C8	1.549	1.536	C23C8N1	111.1	111.7
C8N1	1.476	1.474			
N1C4	1.468	1.482	N1C4C5	113.1	112.2
C4C5	1.524	1.518	C4C5C6	110.1	110.7
C5C6	1.528	1.502	C5C6C7	109.2	110.2
C6C7	1.528	1.509	C6C7C8	112.2	112.5
C7C8	1.535	1.528	C7C8N1	111.1	109.7
C23C27	1.533	1.534	C20C23C27	112.1	111.7
C27N30	1.489	1.46 *	C23C27N30	108.9	-
N30N31	1.225	1.24 *	C27N30N31	115.8	-
N31N32	1.133	1.13 *	N30N31N32	174.1	-
**Dihedral Angle**	**Calc. Dihedral Angle (°) in QAGAP1**	**Exp. Dihedral Angle (°) in Lupinine**	**Dihedral Angle**	**Calc. Dihedral Angle (°) in QAGAP1**	**Exp. Dihedral Angle (°) in Lupinine**
N1C3C2C20	−54.7	−58.1	N1C4C5C6	56.5	56.2
C3C2C20C23	53.4	54.9	C4C5C6C7	−54.8	−52.9
C2C20C23C8	−54.4	−53.6	C5C6C7C8	55.1	54.2
C20C23C8N1	56.3	54.5	C6C7C8N1	−54.7	−56.7
C23C8N1C3	−57.7	−56.5	C7C8N1C4	54.1	57.8
C20C23C27N30	56.7	-			
C23C27N30N31	−177.4	-			
C27N30N31N32	−179.7	-			

* The experimental data for formyl azide were taken from [19] for comparison. The other data were taken from [13].

**Table 2:** The calculated chemical shifts for the carbon and proton nuclei in the QAGAP1, QAGAP1-, QAGAP2 and QAGAP2- structures and in the Boltzmann-averaged at T = 298.15K ensembles of the two lowest-energy (QAGAP1, QAGAP1-) and four lowest-energy (QAGAP1, QAGAP1-, QAGAP2, QAGAP2-) structures in comparison with the experimental data [15]. The assignment of peaks, which differs from that proposed in [15], is given in the last column. The atoms sensitive to the state are highlighted with italic and bold fonts simultaneously.

Calculated Chemical Shifts (in ppm) for Selected Structures and Ensembles								Exp. Chemical Shifts ***	Assignment
QAGAP1	QAGAP1-	QAGAP2	QAGAP2-	Q_1_1-*	Q_1_1- Scaled Carbon Chem. Shifts	Q_1_1-_2_2- **	Q_1_1-_2_2- Scaled Carbon Chem. Shifts		
69.13	69.35	69.46	69.24	69.23	64.50	69.27	64.58	64.3	8-C
61.74	61.76	61.27	61.29	61.75	57.09	61.61	56.88	57.2	3-C
61.27	61.33	60.88	60.91	61.30	56.65	61.18	56.45	57.2	4-C
***54.47***	***54.41***	***57.83***	***57.49***	***54.44***	***49.85***	***55.41***	***50.65***	***50.4***	***27-C***
43.63	43.69	44.81	44.33	43.66	39.17	43.93	39.11	38.2	23-C
34.52	34.58	34.93	35.21	34.55	30.14	34.70	29.83	29.6	7-C
30.57	30.52	30.53	30.49	30.55	26.17	30.54	25.65	25.4	5-C
***30.33***	***30.27***	***36.74***	***36.83***	***30.30***	***25.93***	***32.25***	***27.37***	***27.3***	***20-C***
29.46	29.49	29.34	29.41	29.47	25.10	29.44	24.55	24.9	6-C
24.45	24.61	26.43	26.31	24.52	20.20	25.07	20.16	20.7	2-C
***3.82***	***3.06***	***3.26***	***3.26***	***3.49***	***-***	***3.42***	***-***	***3.42***	***29-H***
***3.70***	***3.57***	***4.14***	***3.31***	***3.64***	***-***	***3.67***	***-***	***3.54***	***28-H***
2.69	2.67	2.64	2.64	2.68	-	2.67	-	2.72–2.82 (2.77)	10-H
2.68	2.67	2.71	2.70	2.68	-	2.68	-	2.72–2.82 (2.77)	12-H
***2.01***	***2.19***	***1.67***	***1.68***	***2.09***	***-***	***1.96***	***-***	***1.80–1.99 (1.89)***	***25-H***
1.98	1.99	1.96	1.98	1.98	-	1.98	-	1.80–1.99 (1.89)	21-H
1.96	2.04	1.89	1.98	1.99	-	1.98	-	1.80–1.99 (1.89)	18-H
1.89	1.88	1.98	1.98	1.89	-	1.92	-	1.80–1.99 (1.89)	11-H
***1.78***	***1.67***	***1.42***	***1.43***	***1.73***	***-***	***1.64***	***-***	***1.58–1.76 (1.67)***	***9-H***
1.70	1.71	1.73	1.73	1.71	-	1.71	-	1.58–1.76 (1.67)	15-H
1.52	1.52	1.54	1.53	1.52	-	1.52	-	1.30–1.57 (1.44)	14-H
***1.46***	***1.78***	***1.30***	***1.76***	***1.60***	***-***	***1.58***	***-***	***1.30–1.57 (1.44)***	***26-H***
1.41	1.41	1.49	1.46	1.41	-	1.43	-	1.30–1.57 (1.44)	13-H
1.34	1.37	1.33	1.41	1.35	-	1.36	-	1.30–1.57 (1.44)	17-H
1.33	1.33	1.34	1.34	1.33	-	1.33	-	1.30–1.57 (1.44)	19-H
***1.32***	***1.34***	***1.52***	***1.66***	***1.33***	***-***	***1.41***	***-***	***1.30–1.57 (1.44)***	***16-H***
***1.22***	***1.31***	***1.47***	***1.59***	***1.26***	***-***	***1.34***	***-***	***1.12–1.26 (1.19)***	***24-H***
1.21	1.21	1.17	1.19	1.21	-	1.20	-	1.12–1.26 (1.19)	22-H

* For brevity, the designation of the ensembles of the two lowest-energy (QAGAP1, QAGAP1-) states; ** for brevity, the designation of the ensembles of the four lowest-energy (QAGAP1, QAGAP1-, QAGAP2, QAGAP2-) states; *** the average value for the given interval is presented in brackets.

## Data Availability

Data may be provided if there is a reasonable request.

## Supplementary Materials

The following supporting information can be downloaded at https://www.mdpi.com/article/10.3390/molecules30030582/s1.
