# Supplementary material for: Arrangement of Azidomethyl Group in Lupinine Azide: Structural and Spectroscopic Properties
Source: Molecules. 2025 Jan 27;30(3):582. doi: 10.3390/molecules30030582 (PMC11820821; doi:10.3390/molecules30030582)
Supplement: Supplementary file 1 [file molecules-30-00582-s001.zip › molecules-3392785-supplementary.pdf]

Supporting information

for

Arrangement of Azidomethyl Group in Lupinine Azide: Structural and Spectroscopic Properties

Kymbat Kopbalina[1,*], Dmitrii Pankin[2], Mikhail Smirnov[3], Niyazbek Ibrayev[4,*], Dastan Turdybekov[5]

[1] Department of Physics and Nanotechnology, Buketov Karaganda University, Universitetskaya 28, 100024 Karaganda, Kazakhstan; kymbatkargtu@gmail.com
[2] Center for Optical and Laser Materials Research, St. Petersburg State University, Ulianovskaya 5, 198504 St. Petersburg, Russia; dmitrii.pankin@spbu.ru
[3] Faculty of Physics, St. Petersburg State University, Universitetskaya Nab. 7/9, 199034 St. Petersburg, Russia; m.smirnov@spbu.ru
[4] Institute of Molecular Nanophotonics, Buketov Karaganda University, Universitetskaya 28, 100024 Karaganda, Kazakhstan; niazibrayev@mail.ru
[5] Department of Physics, Abylkas Saginov Karaganda Technical University, Ave. Nazarbayev 56, 100027 Karaganda, Kazakhstan, turdas@mail.ru
* Correspondence: kymbatkargtu@gmail.com; niazibrayev@mail.ru

1.1 QAGAP1 structure

N	-1.33499600	0.58428900	-0.51537900
C	0.39148900	2.35697300	-0.22580800
C	-1.07560700	2.02840300	-0.47208900
C	-2.76199000	0.37604700	-0.78991700
C	-3.14889100	-1.09616300	-0.85719700
C	-2.75798000	-1.80282200	0.43972400
C	-1.27042700	-1.57611400	0.70542000
C	-0.90484200	-0.08530100	0.72716500
H	0.98717300	2.04707600	-1.08917300
H	-1.40566100	2.45648100	-1.42225100
H	-3.37729500	0.87104500	-0.01345300
H	-2.99307000	0.87196000	-1.73640400
H	-2.63782300	-1.56746100	-1.70317200
H	-4.22367600	-1.17656800	-1.04165000
H	-2.97568800	-2.87275700	0.38357900

H	-0.70374100	-2.08457400	-0.07896400
H	-0.97073500	-2.02108400	1.65891500
H	-1.44209800	0.36699800	1.58542600
H	0.50654900	3.44072300	-0.13821700
C	0.88779200	1.66478100	1.04342500
H	-1.68682800	2.50256200	0.32053600
H	-3.35107500	-1.40089500	1.26941800
C	0.60143500	0.15621200	0.99665700
H	0.36913300	2.08826000	1.91034100
H	1.95441300	1.83989800	1.19396100
H	0.81681900	-0.26730200	1.98352100
C	1.50204800	-0.57024900	-0.00821700
H	1.33326700	-1.64770100	0.03205100
H	1.30179300	-0.23590000	-1.02828000
N	2.92539700	-0.30664100	0.34140900
N	3.75860300	-0.79028600	-0.41513800
N	4.61105200	-1.19041400	-1.04534500

1.2 QAGAP1 calculated vibrational properties.

Mode №	Vibrational mode frequency, cm^{-1}	Vibrational mode scaled* frequency, cm^{-1}	IR intensity, KM/Mole	Raman activity, A4/a.m.u.
1	26.65	25.54	0.0510	10.0328
2	55.56	53.23	0.6889	2.8218
3	91.85	87.99	1.8702	2.3612
4	112.82	108.08	0.8784	2.6759
5	149.01	142.75	1.2600	1.8804
6	192.87	184.77	2.7456	0.3746
7	236.64	226.70	0.6172	1.8296
8	255.24	244.52	3.4870	2.4551
9	295.91	283.48	2.8192	1.3162
10	320.82	307.35	3.6192	0.9124

11	346.77	332.20	0.5715	0.9357
12	357.92	342.89	6.4835	2.0476
13	391.51	375.07	3.0314	2.4322
14	427.43	409.48	0.2909	6.8834
15	479.98	459.83	0.3748	2.4425
16	492.03	471.37	1.3962	4.8904
17	551.31	528.16	0.1925	2.2633
18	570.10	546.16	7.4111	0.9905
19	584.20	559.66	8.7665	0.4863
20	630.18	603.71	5.4210	6.6386
21	681.40	652.78	19.4661	9.4670
22	759.00	727.13	3.3023	12.0337
23	818.05	783.69	6.2990	2.4153
24	830.89	796.00	1.5355	0.6784
25	859.06	822.98	2.5700	1.7898
26	871.10	834.51	2.6006	3.7250
27	885.40	848.22	16.6570	2.3950
28	900.82	862.99	0.8734	12.0248
29	921.02	882.34	4.4401	4.7200
30	944.58	904.91	30.7948	11.8322
31	956.90	916.71	2.7517	3.0776
32	998.41	956.47	0.4012	9.1724
33	1005.36	963.13	7.4449	5.2307
34	1022.32	979.38	6.6637	2.1686
35	1052.82	1008.60	5.6804	5.9657
36	1068.52	1023.64	8.2299	8.5856
37	1079.11	1033.79	11.2338	12.6427
38	1099.02	1052.87	9.7836	1.2605
39	1119.30	1072.29	55.6558	8.4040
40	1125.04	1077.78	8.6252	3.9779
41	1136.96	1089.20	50.7356	2.0599
42	1173.78	1124.48	4.8998	6.0084
43	1196.45	1146.19	0.9574	1.9050
44	1202.18	1151.69	9.8182	2.1049
45	1209.54	1158.74	6.3154	5.2080
46	1249.76	1197.27	5.6955	8.1594
47	1276.78	1223.16	5.8285	20.9239
48	1291.12	1236.89	4.5491	8.5937
49	1299.80	1245.20	16.2299	8.8796
50	1309.79	1254.77	10.3981	32.3650
51	1321.10	1265.61	265.4055	21.9250
52	1328.06	1272.28	20.5536	8.3505
53	1337.53	1281.35	11.1506	5.6872
54	1362.84	1305.61	1.6482	16.5105
55	1367.62	1310.18	12.8517	1.4458
56	1372.17	1314.54	8.1646	1.8162
57	1378.46	1320.57	8.0212	0.9506

58	1380.59	1322.60	2.1202	3.7104
59	1386.67	1328.43	24.4134	3.6432
60	1390.10	1331.72	6.3172	1.4236
61	1403.42	1344.48	11.7227	6.8161
62	1412.57	1353.24	0.9112	1.7781
63	1437.04	1376.69	5.9450	13.8971
64	1477.41	1415.36	3.8092	11.5680
65	1482.94	1420.66	2.7396	18.2723
66	1486.09	1423.68	7.2147	13.0969
67	1488.06	1425.56	17.1428	3.9133
68	1493.04	1430.33	3.2725	11.0105
69	1498.77	1435.82	8.7997	4.2632
70	1507.81	1444.48	19.3114	23.1998
71	1509.17	1445.79	1.7676	2.8163
72	2203.49	2110.94	985.6956	92.6580
73	2855.11	2735.19	61.7886	89.1919
74	2873.70	2753.01	17.3308	20.5883
75	2882.12	2761.07	279.1998	419.6652
76	3006.61	2880.33	38.8030	224.7118
77	3012.72	2886.19	13.8118	139.8767
78	3017.88	2891.13	32.4735	121.4719
79	3023.53	2896.55	28.4080	26.6594
80	3026.02	2898.92	72.0624	391.0220
81	3027.39	2900.24	35.8704	92.0031
82	3042.68	2914.89	17.1385	243.8457
83	3045.90	2917.97	15.0829	37.5323
84	3049.65	2921.56	139.2154	317.0686
85	3053.64	2925.39	56.4157	467.6472
86	3059.48	2930.99	61.7606	185.5911
87	3062.18	2933.57	50.9332	180.4838
88	3067.21	2938.38	126.4483	74.2558
89	3077.31	2948.07	55.6490	223.0298
90	3086.52	2956.88	30.6580	81.9346

*the scaling factor is 0.958

2.1. QAGAP1- structure

N -1.46814000 0.63750400 -0.33484600

C 0.19410000 2.47765300 -0.08705700

C -1.27118000 2.06387200 -0.04604100

C -2.90833100 0.35163700 -0.30172300

C -3.23416800 -1.10965200 -0.58327200

C	-2.49915000	-2.01220700	0.40611500
C	-1.00392000	-1.70328700	0.35093300
C	-0.71087900	-0.21666600	0.59960100
H	0.56795300	2.40608100	-1.11224500
H	-1.84704000	2.63558400	-0.77850900
H	-3.32978100	0.62655400	0.68470300
H	-3.39111200	0.99719900	-1.04009200
H	-2.93237200	-1.35932900	-1.60578300
H	-4.31616300	-1.25422800	-0.52003200
H	-2.67823300	-3.06716400	0.18201300
H	-0.63222500	-1.99384700	-0.63503600
H	-0.45491100	-2.29450800	1.08990800
H	-1.03387500	0.00765500	1.63678000
H	0.27785000	3.52831000	0.20376200
C	1.02502400	1.60020500	0.84910100
H	-1.68689200	2.31178000	0.95007500
H	-2.87920900	-1.83262100	1.41842600
C	0.80270700	0.10970000	0.54940600
H	0.72476700	1.79390000	1.88437600
H	2.08552900	1.84756100	0.77357200
H	1.27197700	-0.47816700	1.34682000
C	1.45850700	-0.31447300	-0.77756300
H	1.33515000	-1.38427100	-0.94983900
H	1.01088800	0.21004100	-1.61771500
N	2.90869100	0.01521500	-0.83463400
N	3.65687800	-0.73884700	-0.22181700
N	4.44420500	-1.36833300	0.29584300

2.2. QAGAP1- calculated vibrational properties.

Mode №	Vibrational mode frequency, cm^{-1}	Vibrational mode scaled* frequency, cm^{-1}	IR intensity, KM/Mole	Raman activity, A4/a.m.u.
1	31.51	30.18	0.1472	7.2764
2	34.37	32.93	0.9754	4.1492
3	78.07	74.79	0.2503	2.8539
4	128.30	122.91	0.2644	1.1012
5	157.44	150.83	1.4229	1.5839
6	207.68	198.95	1.7187	1.1192
7	224.94	215.49	2.7429	2.1689
8	287.88	275.79	4.7804	1.6406
9	304.66	291.87	1.5435	1.9642
10	315.43	302.19	2.6858	0.5483
11	348.15	333.53	0.7581	0.6084
12	352.22	337.43	6.5247	2.5207
13	387.13	370.87	2.0337	1.5658
14	427.25	409.31	0.1596	7.7791
15	479.82	459.67	0.8460	1.5493
16	488.48	467.96	0.2879	4.2865
17	549.93	526.83	0.5420	1.7792
18	566.14	542.37	1.9273	0.6245
19	586.17	561.55	13.7010	0.3061
20	645.57	618.46	3.3643	1.5186
21	702.90	673.38	22.7130	6.3444
22	746.18	714.84	8.3541	17.7463
23	809.63	775.62	4.6623	3.6223
24	829.29	794.46	0.7398	1.4019
25	857.13	821.13	3.7594	2.2402
26	871.25	834.66	7.4058	2.9376
27	884.09	846.96	6.5503	3.2307
28	898.81	861.06	1.1875	13.5589
29	902.06	864.17	28.0470	10.9585
30	932.44	893.28	17.0500	1.8155
31	959.47	919.17	1.1342	2.4343
32	996.30	954.46	1.3639	4.8276
33	1005.57	963.33	7.4903	7.3551
34	1026.68	983.56	8.0534	3.6478
35	1044.17	1000.31	7.7436	5.9703
36	1067.73	1022.89	10.5773	7.8583
37	1077.86	1032.59	14.9411	14.3855
38	1097.85	1051.74	11.4054	1.3652
39	1118.69	1071.71	39.3153	6.9530
40	1127.31	1079.96	16.0524	4.8124

41	1135.48	1087.79	44.5159	1.5300
42	1173.37	1124.09	6.6766	5.4552
43	1197.44	1147.14	1.2188	1.2609
44	1202.41	1151.91	9.9012	2.4468
45	1216.19	1165.11	4.6297	6.9953
46	1253.05	1200.42	14.0307	13.4122
47	1277.36	1223.71	2.3026	19.5953
48	1289.41	1235.26	13.0224	7.1241
49	1298.49	1243.95	25.0930	12.9568
50	1308.16	1253.22	148.0752	13.1862
51	1309.18	1254.20	31.3629	26.6303
52	1337.72	1281.54	14.9519	5.3859
53	1343.78	1287.34	62.6793	9.1837
54	1359.18	1302.10	6.5279	15.0560
55	1367.90	1310.45	3.5963	1.7125
56	1372.81	1315.15	6.0158	1.9560
57	1377.80	1319.93	2.9684	1.2847
58	1381.48	1323.46	1.9466	4.7196
59	1385.31	1327.13	22.6674	2.6893
60	1388.87	1330.54	4.0516	1.7658
61	1401.55	1342.69	9.3754	7.2960
62	1407.52	1348.41	13.6072	4.5113
63	1434.58	1374.33	8.2813	11.5887
64	1476.74	1414.72	4.1967	11.8589
65	1482.09	1419.84	3.2826	24.9892
66	1484.70	1422.34	11.3713	4.9713
67	1486.32	1423.89	12.6817	6.8296
68	1490.78	1428.17	0.1888	10.2638
69	1496.76	1433.90	3.8883	1.9961
70	1507.25	1443.95	12.4418	30.0190
71	1509.33	1445.94	2.1741	7.2706
72	2195.58	2103.37	926.2083	76.5966
73	2850.98	2731.24	61.9792	87.6153
74	2874.48	2753.75	17.5439	23.1726
75	2882.72	2761.65	268.8079	399.2390
76	3006.58	2880.30	34.1176	73.9409
77	3007.23	2880.93	21.7404	226.7518
78	3017.94	2891.18	33.9838	115.8741
79	3020.90	2894.03	49.0021	217.8087
80	3024.83	2897.79	49.0890	172.7489
81	3027.82	2900.65	31.3661	121.4174
82	3045.34	2917.43	7.5948	28.2955
83	3049.60	2921.52	155.2682	283.0881
84	3053.95	2925.68	61.9218	534.4281
85	3058.49	2930.04	25.1180	107.2909
86	3061.99	2933.39	79.4016	234.9071
87	3062.45	2933.83	39.2497	135.4181

88	3072.37	2943.33	83.1368	126.7492
89	3074.30	2945.18	66.2879	227.9878
90	3134.39	3002.75	16.4959	72.8998

3.1. QAGAP2 structure

N	-1.41104000	0.25539500	-0.51898200
C	-0.71613800	2.64523300	-0.47693400
C	-1.86072700	1.64872400	-0.61094100
C	-2.56593400	-0.62707500	-0.72412500
C	-2.20127700	-2.10415800	-0.65767400
C	-1.53771200	-2.41827300	0.68119400
C	-0.33831100	-1.49310400	0.88355800
C	-0.71225400	-0.01079400	0.75264000
H	-0.05330800	2.55983400	-1.34359100
H	-2.36231500	1.77676200	-1.57366700
H	-3.34841700	-0.41904700	0.03134100
H	-2.99843900	-0.38540200	-1.69868600
H	-1.51615200	-2.34674400	-1.47671900
H	-3.10412700	-2.70426600	-0.80107100
H	-1.22089600	-3.46383900	0.72565700
H	0.42798300	-1.74278500	0.14580300
H	0.11277500	-1.64970600	1.86725000
H	-1.38817800	0.23525800	1.59636600
H	-1.11982300	3.66124400	-0.48664200
C	0.05991400	2.38700300	0.81471500
H	-2.61459000	1.86044700	0.17225500
H	-2.26304800	-2.27253500	1.49048200
C	0.51705000	0.92175000	0.89199600

H	-0.58941400	2.59741000	1.67136500
H	0.91979000	3.05764200	0.89756000
H	0.95141500	0.74576300	1.88032600
C	1.61920500	0.64172900	-0.14225700
H	1.18801700	0.26543200	-1.07287000
H	2.16389100	1.56338300	-0.36804400
N	2.58962500	-0.34963700	0.40284200
N	3.49036800	-0.68430500	-0.35821800
N	4.36418100	-1.06217700	-0.97280500

3.2. QAGAP2 calculated vibrational properties

Mode №	Vibrational mode frequency, cm^{-1}	Vibrational mode scaled* frequency, cm^{-1}	IR intensity, KM/Mole	Raman activity, A4/a.m.u.
1	24.64	23.60	0.0853	9.9695
2	64.90	62.18	0.3750	2.7820
3	100.55	96.32	1.8114	3.6746
4	115.98	111.11	3.8657	0.8682
5	145.22	139.12	0.5196	2.5327
6	187.42	179.54	1.5929	3.4089
7	229.02	219.40	2.3778	1.2179
8	259.70	248.79	0.4763	0.8706
9	300.02	287.42	0.6471	2.4519
10	327.19	313.45	3.5641	1.4394
11	335.44	321.35	1.0783	0.5822
12	377.69	361.83	3.2135	2.4547
13	396.64	379.98	14.6541	0.9827
14	421.67	403.96	0.2678	4.5688
15	455.99	436.84	0.8182	5.0619
16	499.70	478.72	1.2205	7.2276
17	549.49	526.41	0.0444	1.0523
18	555.09	531.78	0.5563	3.7401
19	583.79	559.27	10.6410	0.3036
20	630.61	604.12	4.4006	5.7424
21	688.18	659.28	22.3288	5.9173
22	764.32	732.22	5.1570	10.5677
23	828.25	793.46	3.6695	1.7637
24	850.38	814.66	1.2854	2.0742
25	857.34	821.33	3.6027	0.9988

26	885.38	848.20	0.5063	9.7996
27	890.18	852.79	10.9989	5.1191
28	905.30	867.28	8.7376	5.5760
29	920.63	881.96	2.4677	8.2267
30	936.07	896.75	7.6913	7.9188
31	946.14	906.40	13.7858	9.2247
32	994.57	952.80	2.3039	1.4148
33	1008.12	965.78	5.2217	5.9629
34	1016.07	973.40	8.4641	0.6553
35	1051.05	1006.90	7.9761	3.5818
36	1066.13	1021.35	8.5232	7.9419
37	1074.30	1029.18	11.8411	16.0159
38	1099.97	1053.77	9.3792	1.5391
39	1120.39	1073.33	53.0485	7.1517
40	1124.34	1077.12	11.5828	4.8071
41	1137.92	1090.12	48.6485	1.4440
42	1172.43	1123.18	4.4015	5.9789
43	1195.23	1145.03	0.0518	1.2034
44	1207.35	1156.64	8.2424	2.0144
45	1219.51	1168.29	2.0452	6.1158
46	1241.37	1189.23	11.9102	9.9140
47	1275.32	1221.76	10.6841	19.2946
48	1289.46	1235.30	5.4147	8.4690
49	1297.83	1243.32	7.1133	28.8046
50	1307.15	1252.25	54.5314	17.2823
51	1315.73	1260.47	219.4392	26.1098
52	1318.01	1262.66	26.4585	5.6228
53	1342.08	1285.71	2.8997	3.7063
54	1358.82	1301.75	10.9803	8.0290
55	1366.56	1309.16	11.4751	2.1608
56	1373.10	1315.43	4.6093	1.1598
57	1378.29	1320.40	12.3876	4.2511
58	1380.33	1322.36	1.4364	2.7398
59	1385.50	1327.31	6.1470	3.1433
60	1387.00	1328.75	22.4872	2.7070
61	1402.09	1343.20	3.5898	4.1888
62	1412.55	1353.23	10.3022	3.3962
63	1435.52	1375.23	3.4448	12.3374
64	1474.89	1412.95	3.4797	10.9581
65	1485.04	1422.67	4.5239	20.2920
66	1487.51	1425.03	18.7125	14.6869
67	1488.82	1426.29	18.2420	3.9229
68	1493.37	1430.65	0.4457	16.4426
69	1500.17	1437.16	16.5915	2.4953
70	1501.26	1438.21	6.5586	7.2228
71	1509.23	1445.84	5.9027	15.2379
72	2200.02	2107.61	960.7286	87.7941

73	2858.35	2738.30	65.2447	91.3613
74	2875.49	2754.72	14.6378	21.9113
75	2883.48	2762.37	294.2591	447.0315
76	3003.21	2877.07	43.8799	217.5659
77	3012.65	2886.12	27.8262	105.6851
78	3017.01	2890.29	39.1130	233.7348
79	3017.49	2890.75	26.4789	150.3833
80	3022.18	2895.25	31.9205	191.1951
81	3031.05	2903.75	20.9715	82.6911
82	3039.27	2911.62	51.6500	260.3451
83	3044.03	2916.18	27.0336	39.5267
84	3048.91	2920.86	104.5842	209.0528
85	3051.92	2923.74	130.7275	66.3587
86	3053.61	2925.36	28.4382	796.2490
87	3058.79	2930.32	64.1853	172.1124
88	3062.12	2933.51	78.4900	135.0503
89	3066.66	2937.86	24.0829	79.2742
90	3072.80	2943.75	87.4384	89.4203

4.1. QAGAP2- structure

N -1.51013300 0.43933000 -0.32435500

C -0.48960000 2.70630600 -0.20165500

C -1.75786900 1.86784300 -0.09923900

C -2.79098200 -0.27578000 -0.27222900

C -2.64366600 -1.77415900 -0.50165600

C -1.67456500 -2.36690900 0.51916200

C -0.34627600 -1.61507000 0.45124900

C -0.52116800 -0.10140100 0.62783000

H -0.12608500 2.69531300 -1.23361000

H -2.49166700 2.20126000 -0.83753300

H -3.28384000 -0.11188700 0.70588600

H -3.44556500 0.16251600 -1.03032400

H -2.26807300 -1.95161500 -1.51475700

H -3.62767400 -2.24625400 -0.43261000

H -1.51656700 -3.43316400 0.33618200

H	0.12720200	-1.81892700	-0.51196800
H	0.34222500	-1.97428600	1.22159600
H	-0.88349700	0.06990700	1.66183400
H	-0.72469900	3.74635600	0.03962900
C	0.57944000	2.16451800	0.74726500
H	-2.21350500	2.02618500	0.89772300
H	-2.10411700	-2.27751100	1.52412400
C	0.81838400	0.66565700	0.50292100
H	0.24504800	2.30225700	1.78109400
H	1.51565300	2.71979400	0.64213600
H	1.47878400	0.29505500	1.29423100
C	1.53129600	0.44908200	-0.84953300
H	0.80355700	0.33866400	-1.64959900
H	2.16046600	1.31323500	-1.08349500
N	2.37115800	-0.77647700	-0.92243400
N	3.36684600	-0.79368200	-0.20773300
N	4.31247100	-0.92308700	0.40377500

4.2. QAGAP2- calculated vibrational properties

Mode №	Vibrational mode frequency, cm^{-1}	Vibrational mode scaled* frequency, cm^{-1}	IR intensity, KM/Mole	Raman activity, A4/a.m.u.
1	27.67	26.51	0.4404	6.9239
2	56.86	54.47	0.6995	3.9951
3	95.13	91.13	0.9494	3.2863
4	124.17	118.95	0.6447	1.2569
5	163.24	156.38	0.5763	0.3462
6	188.31	180.40	2.2071	4.2364
7	254.18	243.50	1.2886	0.5760
8	265.79	254.63	6.4842	2.0402
9	314.73	301.51	2.2522	0.5873
10	332.60	318.63	2.1999	0.6924

11	343.11	328.70	0.2745	1.2609
12	378.42	362.52	1.5933	1.8386
13	392.84	376.34	10.4835	2.3005
14	422.30	404.56	0.1321	4.4291
15	455.70	436.56	0.8848	5.4247
16	500.47	479.45	0.5336	7.4381
17	548.93	525.87	0.4021	2.3487
18	555.31	531.99	1.0942	2.8018
19	586.24	561.62	12.6008	0.3073
20	645.33	618.22	4.7713	1.3062
21	694.79	665.61	20.7980	2.6741
22	761.37	729.40	9.2361	13.6533
23	826.37	791.66	2.3931	4.0911
24	847.41	811.82	1.2802	2.3929
25	859.71	823.60	3.6655	1.5563
26	880.46	843.48	3.2886	4.5612
27	890.15	852.77	2.9424	16.1340
28	898.05	860.33	23.0887	8.1838
29	907.40	869.29	2.4684	5.3625
30	936.10	896.78	11.5366	3.3482
31	944.62	904.94	9.7313	5.2064
32	989.69	948.12	4.0660	3.8249
33	1008.53	966.17	6.8828	4.0321
34	1018.72	975.93	5.5386	0.7993
35	1047.20	1003.22	7.3513	5.2475
36	1068.26	1023.39	9.4628	6.2682
37	1073.73	1028.64	10.6977	17.1863
38	1101.07	1054.83	12.4083	1.5902
39	1120.67	1073.60	48.6464	8.3157
40	1123.99	1076.78	8.7597	3.8820
41	1138.79	1090.96	48.2259	1.0858
42	1170.65	1121.48	6.3221	5.1825
43	1193.78	1143.64	0.2636	1.0689
44	1207.52	1156.81	8.6614	2.8233
45	1219.68	1168.46	4.9772	6.7591
46	1251.55	1198.98	5.0615	16.6473
47	1275.37	1221.80	4.4353	17.1145
48	1289.12	1234.98	5.9044	11.4253
49	1299.65	1245.07	37.5206	21.6549
50	1303.59	1248.84	209.8939	12.5336
51	1312.85	1257.71	19.6204	17.6784
52	1323.17	1267.59	12.3731	6.2540
53	1347.58	1290.98	31.3668	7.1586
54	1360.82	1303.66	8.8649	5.3301
55	1369.16	1311.66	1.4146	3.1768
56	1373.18	1315.50	2.4918	0.8445
57	1379.10	1321.18	1.1399	11.1891

58	1380.67	1322.68	2.7439	1.8088
59	1386.52	1328.29	10.0637	3.9437
60	1387.55	1329.28	22.6861	2.8280
61	1403.66	1344.71	8.3568	3.2786
62	1409.54	1350.34	8.8671	4.8973
63	1434.35	1374.11	6.2944	11.2126
64	1477.55	1415.49	4.8196	13.6661
65	1485.59	1423.19	3.1689	24.6830
66	1487.08	1424.62	16.3061	7.5979
67	1488.87	1426.34	9.7797	7.0724
68	1494.62	1431.84	1.3006	16.3085
69	1500.71	1437.68	2.6449	3.3403
70	1505.70	1442.46	9.2682	10.3000
71	1508.85	1445.48	6.3634	16.1286
72	2193.89	2101.75	881.3124	72.8495
73	2856.10	2736.14	62.8669	71.6252
74	2874.19	2753.48	18.1999	28.4407
75	2883.19	2762.10	278.8789	434.5744
76	3002.98	2876.86	42.9839	205.5100
77	3011.14	2884.67	19.3447	109.9867
78	3017.05	2890.33	31.4473	126.3045
79	3022.66	2895.71	53.8565	49.8994
80	3024.60	2897.57	18.9357	74.4425
81	3024.81	2897.77	35.7273	338.4727
82	3031.39	2904.07	56.9435	222.5262
83	3043.80	2915.96	19.5806	45.9952
84	3049.32	2921.25	114.4334	245.7404
85	3052.59	2924.38	130.1606	78.9793
86	3054.20	2925.92	31.2060	730.6630
87	3058.80	2930.33	64.0279	187.0546
88	3063.79	2935.11	73.7925	142.7448
89	3074.30	2945.18	63.8592	90.1321
90	3126.48	2995.17	10.1758	71.8091
